# Infectious laryngotracheitis: Etiology, epidemiology, pathobiology, and advances in diagnosis and control – a comprehensive review

**DOI:** 10.1080/01652176.2020.1759845

**Published:** 2020-05-04

**Authors:** Vasudevan Gowthaman, Sachin Kumar, Monika Koul, Urmil Dave, T. R. Gopala Krishna Murthy, Palanivelu Munuswamy, Ruchi Tiwari, Kumaragurubaran Karthik, Kuldeep Dhama, Izabela Michalak, Sunil K. Joshi

**Affiliations:** a Poultry Disease Diagnosis and Surveillance Laboratory, Tamil Nadu Veterinary and Animal Sciences University, Namakkal, Tamil Nadu, India; b Department of Biosciences and Bioengineering, Indian Institute of Technology Guwahati, Guwahati, Assam, India; c Division of Pathology, ICAR – Indian Veterinary Research Institute, Izatnagar, Uttar Pradesh, India; d Department of Veterinary Microbiology and Immunology, College of Veterinary Sciences, UP Pandit Deen Dayal Upadhayay Pashu Chikitsa Vigyan Vishwavidyalay Evum Go-Anusandhan Sansthan (DUVASU), Mathura, Uttar Pradesh, India; e Central University Laboratory, Tamil Nadu Veterinary and Animal Sciences University, Chennai, Tamil Nadu, India;; f Faculty of Chemistry, Department of Advanced Material Technologies, Wrocław University of Science and Technology, Wrocław, Poland; g Department of Microbiology & Immunology, Department of Pediatrics, Division of Hematology, Oncology and Bone Marrow Transplantation, University of Miami School of Medicine, Miami, Florida, USA

**Keywords:** poultry, chicken, Infectious Laryngotracheitis virus, ILT, epidemiology, pathobiology, diagnosis, vaccine, control, review

## Abstract

Infectious laryngotracheitis (ILT) is a highly contagious upper respiratory tract disease of chicken caused by a Gallid herpesvirus 1 (GaHV-1) belonging to the genus *Iltovirus,* and subfamily *Alphaherpesvirinae* within *Herpesviridae* family. The disease is characterized by conjunctivitis, sinusitis, oculo-nasal discharge, respiratory distress, bloody mucus, swollen orbital sinuses, high morbidity, considerable mortality and decreased egg production. It is well established in highly dense poultry producing areas of the world due to characteristic latency and carrier status of the virus. Co-infections with other respiratory pathogens and environmental factors adversely affect the respiratory system and prolong the course of the disease. Latently infected chickens are the primary source of ILT virus (ILTV) outbreaks irrespective of vaccination. Apart from conventional diagnostic methods including isolation and identification of ILTV, serological detection, advanced biotechnological tools such as PCR, quantitative real-time PCR, next generation sequencing, and others are being used in accurate diagnosis and epidemiological studies of ILTV. Vaccination is followed with the use of conventional vaccines including modified live attenuated ILTV vaccines, and advanced recombinant vector vaccines expressing different ILTV glycoproteins, but still these candidates frequently fail to reduce challenge virus shedding. Some herbal components have proved to be beneficial in reducing the severity of the clinical disease. The present review discusses ILT with respect to its current status, virus characteristics, epidemiology, transmission, pathobiology, and advances in diagnosis, vaccination and control strategies to counter this important disease of poultry.

## 1. Introduction

Poultry farming is one of the rapidly developing sectors, which plays an important role in the global food security. The consequence of globalization, climate change and rapidly expanding poultry population results in the emergence of several diseases. Among the emerging diseases, infectious laryngotracheitis (ILT) is a highly contagious upper respiratory tract disease of chicken and has been regarded as a major concern for poultry health and welfare (Bagust et al. 2000). Although chickens are considered to be the primary target host (Bagust 1986), natural disease has been reported in peafowls and pheasants (Crawshaw and Boycott 1982; Hanson and Bagust 1991). Other species, including closely related Galliformes are refractory to infection, and birds such as crows, ducks, pigeons, sparrows and starlings seem to be resistant (Guy and Garcia 2008). This disease causes production losses due to increased morbidity, moderate mortality, decreased weight gain, reduced egg production and expenses spent on vaccination, biosecurity measures and therapy to counteract secondary infection by other avian pathogens (Guy and Bagust 2003; Guy and Garcia 2008; Jones 2010; Garcia et al. 2014). In chickens, two main forms of ILT have been described under field conditions which include the severe acute or epizootic form characterized by significant respiratory distress, sneezing, expectoration of blood-mixed mucus, severe haemorrhagic tracheitis and conjunctivitis accompanied by high mortality reaching up to 70% (ranging from 5 to 70%) and a milder form characterized by mild to moderate catarrhal tracheitis, sinusitis, conjunctivitis, relatively low morbidity and occasional mortality which usually range between 0.1 and 2% (Ou and Giambrone 2012). Chicken embryo origin (CEO) and tissue culture origin (TCO) vaccines developed during 1960s have been extensively used for controlling ILT outbreaks worldwide. In the meantime, both the vaccines had the tendency to revert to virulence following bird to bird passages. It is believed that most of the outbreaks are caused by CEO vaccine isolates that persist in long-lived bird operations and spill-over into poultry populations (Blacker et al. 2011). The recombinant/mutant vaccines, which are considered to be safer alternatives, have limited practical applicability because they fail to stop complete viral shedding and existence of antibodies against vectors can neutralise the vaccines. Increased incidence of the disease is due to more concrete factors such as increase in poultry production density, decrease in downtime of production sites, poor biosecurity, and poor vaccination methods. Vaccine virus reactivation and shedding has been reported from several parts in commercial layers (Thilakarathne et al. 2020). Hence, serious attention must be given to control the ILT in poultry-dense areas not only to prevent the economic loss but also to enhance the poultry welfare and health.

The present review focuses on the comprehensive overview of the ILT with respect to its etiology, epidemiology, transmission, pathobiology, advances in diagnosis and vaccines, and appropriate prevention and control strategies.

## 2. Etiology

### 2.1. The virus

**Figure 1.**
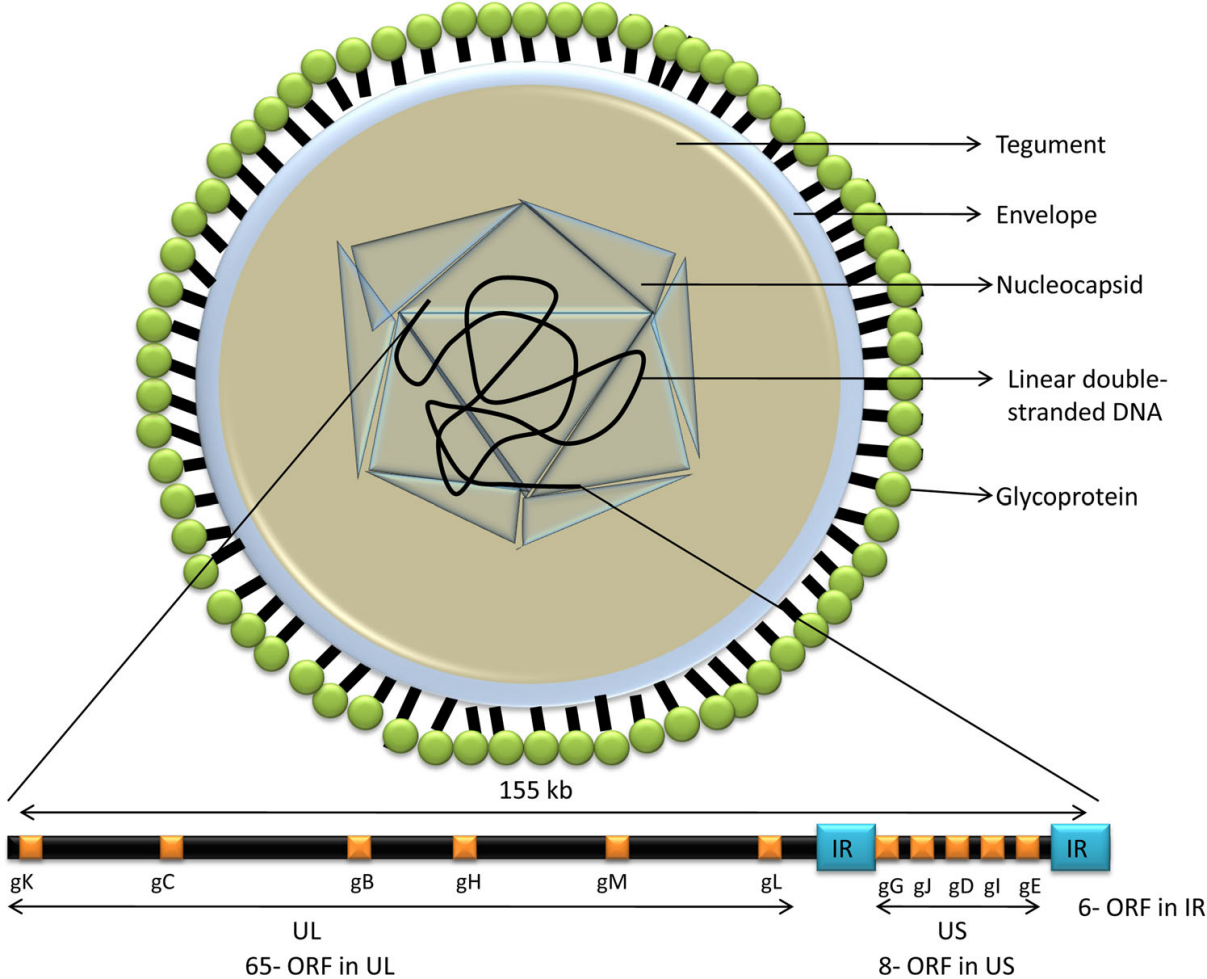
Structure of ILT virus.

ILT is caused by the infectious laryngotracheitis virus, also known as Gallid herpesvirus 1 (GaHV-1), which belongs to the genus *Iltovirus*, subfamily *Alphaherpesvirinae* of the family *Herpesviridae* (Davison et al. 2009). The genome of ILTV contains a 150-155 kb linear double-stranded DNA encoding a unique long (UL), unique short (US) and two inverted repeat (IR) sequences (Figure 1) (McGeoch et al. 2000; Morales Ruiz et al. 2018). A fully assembled complete genome sequence of ILTV comprises 148 kb nucleotides, with a G + C content of 48.2% (Lee et al. 2011). The virions of ILTV under electron microscopy appear as typical herpes virions consisting of a DNA core within an icosahedral capsid which is surrounded by a tegument layer, and outer envelope glycoproteins (Roizman and Pellett 2001). The size of the viral capsid is about 100 nm in diameter, and the complete viral particle size is within the range of 200 to 350 nm (Granzow et al. 2001). The ILTV genome consists of 80 open reading frames (ORFs); out of which 65 are located in the UL region, 9 in the US region and 6 in the IR region (McGeoch et al. 2000; Thureen and Keeler 2006; Lee et al. 2011). Among 80 ORFs, sixty-three ORFs display homologies to Herpes Simplex Virus-1 (HSV-1) genome with respect to position and structure of the deduced translation products. The envelope contains glycoproteins namely gB, gC, gD, gE, gG, gH, gI, gJ, gK, gL and gM, which are encoded by highly conserved ORFs *viz.* UL27, UL44, US6, US8, US4, UL22, US7, US5, UL53, UL1 and UL10, respectively (Piccirillo et al. 2016). The viral glycoproteins are important for ILTV replication and eliciting humoral and cell-mediated immune responses in the host (Roizman and Pellett 2001). There are two clusters of *Iltovirus* specific genes, one is located between UL45 and UL22 which encodes five ORFs (ORF A-E). The second cluster of *Iltovirus* specific genes is located between UL-1 and ICP4 and code for UL-0 and UL-1 (Fuchs and Mettenleiter 1996). The other differing features in ILTV genome are absence of an UL16 or its homologue (Roizman and Knipe 2001), localization of UL47 between the US3 and US4 genes within the US region instead of being located within the UL region and internal inversion of a conserved gene cluster within the UL region (McGeoch et al. 1988; Wild et al. 1996). Two regions designated as UL0 and UL (-1), specific to ILTV genome, show noticeable similarities in the deduced amino acid sequences, suggesting a duplication event during virus evolution (Thureen and Keeler 2006). Deletion of UL (-1) gene of ILTV and replacing with the gene encoding green fluorescent protein (GFP) and major immediate promoter element of cytomegalovirus resulted in defective ILTV which was unable to propagate in permissive cells. Thus, the UL (-1) gene has an important role in ILTV replication (Nadimpalli et al. 2017). Like other alphaherpesviruses, the ILTV genome contains three origins of DNA replication, an OriL positioned within the UL region, and two copies of OriS located within the internal repeat (IR) and terminal repeat (TR) regions (Lee et al. 2011). The ORFs vary in their characteristics from other alphaherpesviruses (McGeoch et al. 2006). The tegument proteins help in the transportation of capsid into the cytoplasm and further to the nucleus (Kelly et al. 2009).

Recent advances in molecular techniques enabled rapid identification of genetic variations with precision. Next generation sequencing platforms such as hybrid next generation sequencing (h-NGS) has been found to be useful to identify mutations in genes related to high and low virulence. Garcia et al. (2013) determined the genomic sequences of low and high passage vaccine strains of ILTV, CEO and TCO by h-NGS.

Virus replication and recombination are near inseparable and hence diverse progeny of recombinant ILT viruses emerge out upon co-infection in natural animal host. Based on TaqMan SNP genotyping assay, 11 SNPs within genes UL (-1), US5, US6, US7, US8, US9 and two SNPs in UL43 and UL47 genes were identified confirming high rate of recombination (Loncoman et al. 2017). ILTV, irrespective of either attenuated strain or wild type, upon infecting the target host, replicate, gain or regain virulence to cause disease, and establishes latent infection. Genome level comparison of field strains of ILTV from different countries with commercially used vaccine strains showed that there were only few amino acids in the field strain similar to vaccine strains. This denotes that field strains might have originated from vaccine strain (Garcia and Spatz 2014).

### 2.2. Viral replication

The replication of ILTV occurs during the first week of infection (Bagust 1986; Williams et al. 1992). Conjunctiva and tracheal mucosa are the major sites of ILTV replication leading to inflammation, serous or mucoid discharge, and respiratory distress (Coppo et al. 2013; Coppo et al. 2013). As ILTV first interacts with the cells lining the nasal cavity, conjunctival mucosa and harderian glands, these tissues play a pivotal role in early virus replication and dictate the fate of infection (Beltrán et al. 2017). Within respiratory system, the epithelial cells that lines larynx and trachea are always affected, while respiratory sinuses, air sacs and lung tissues may or may not be affected (Hanson and Bagust 1991). ILTV can invade the basement membrane of tracheal and conjunctival mucosa in a time dependant manner which promotes virus spread (Reddy et al. 2014). The virus has the ability to establish latency in the trigeminal ganglion during the lytic phase of infection. The ILTV gets reactivated once carrier birds are subjected to stressors such as vaccination, shifting, and during onset of lay. In addition, the ILTV has been detected in other organs, such as the brain, tongue, thymus, lung, heart, proventriculus, pancreas, duodenum, small intestine, large intestine, cecum, cecal tonsils, liver, spleen, kidney, and bursa (Zhao et al. 2013; Wang et al. 2013). These findings raised speculations that the ILTV undergoes systemic replication. Both the vaccine and virulent strains of ILTV could replicate in embryonated chicken neural stem cell; however, cytopathic effects (CPE) such as cell rounding, syncytium formation and cell detachment have been reported in cells infected with vaccine strains of ILTV, but not in cells infected with field virulent strains (Shahsavandi et al. 2017). Increasing numbers of viral nucleic acid in the host cell during virus replication results in accumulation of more viral DNA, subsequently that is incorporated into newly formed viral particles located inside the host nucleus. This leads to development of basophilic intra nuclear inclusion bodies, which can be detected as early as 12 hours of post infection (Reynolds et al. 1968). In the natural host, the replication rate and transmission efficiency has been found to be greater for CEO than TCO strains, and hence the CEO revertant causes a more severe respiratory disease and higher mortality than those caused by TCO revertant (García 2016).

**Figure 2.**
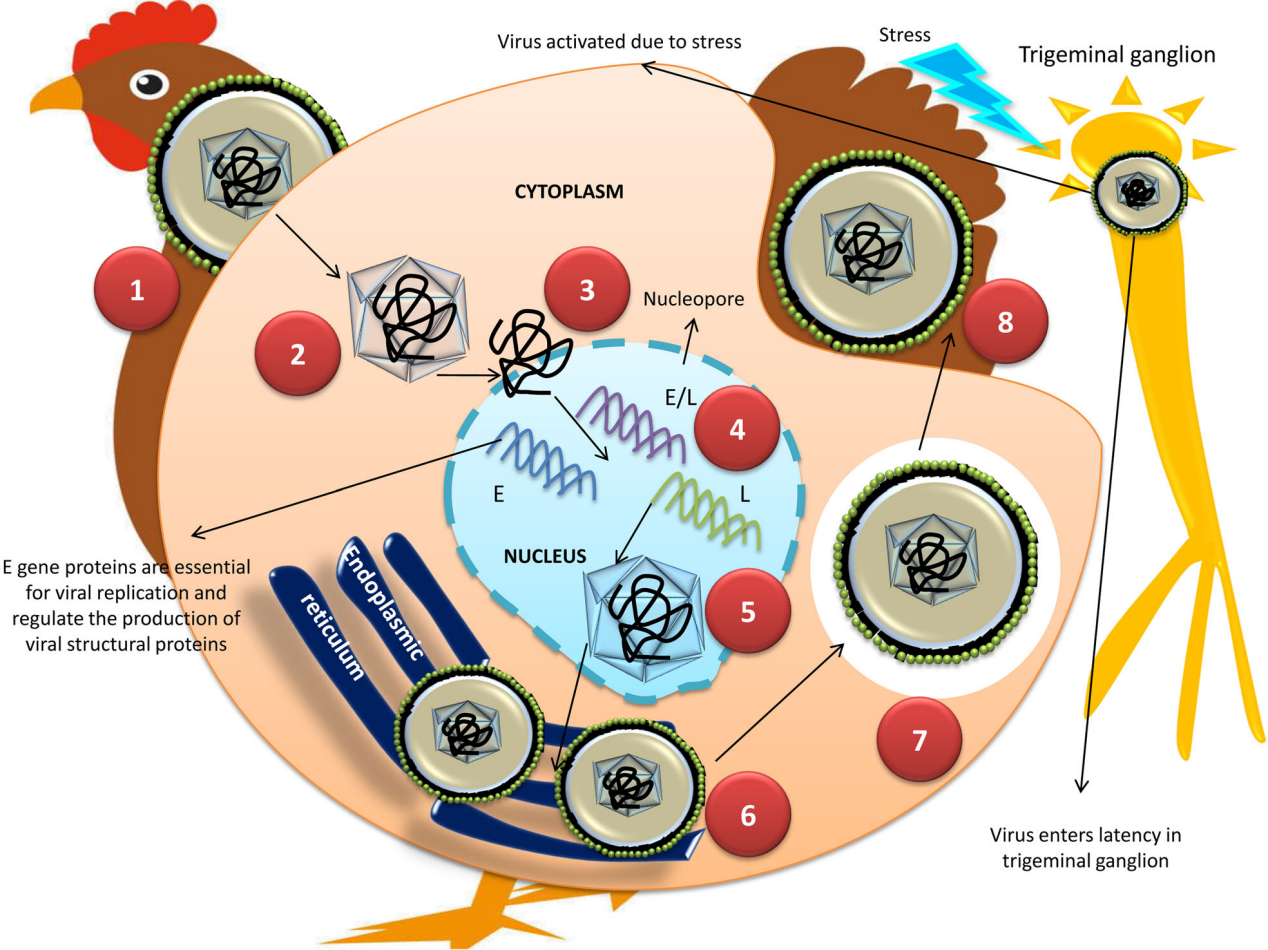
Replication of ILT virus. 1. Attachment 2.Tegument and nucleocapsid get transported into the cytoplasm 3. Viral DNA released from the nucleocapsid enter into the nucleus through nuclear pores 4. Three classes of genes, namely early (E), early/late (E/L) and late (L) are expressed during the viral transcription and translation process based on the levels of expression. 5. Nucleocapsids containing DNA acquire an envelope while budding out from the inner lamellae of the nuclear membrane 6. Virions are transported into the lumen of the endoplasmic reticulum to acquire second envelope and further accumulate within the cytoplasmic vacuoles. 7,8. The vacuoles containing the virions are released out by exocytosis or cell lysis.

The replication mechanism of ILTV seems similar to other alphaherpesviruses such as HSV-1 (Figure 2). Envelope glycoproteins mainly gC, rather than gB, gD, gH, and gL are assumed to mediate the attachment with host cell receptors (Kingsley et al. 1994; Kingsley and Keeler 1999) and helps in the fusion of the viral envelope to the host cell membrane. The entry of ILTV is heparin sulphate independent, unlike HSV-1 (Kingsley and Keeler 1999). After attachment, the tegument and nucleocapsid get transported into the cytoplasm and the viral DNA released from the nucleocapsid enter into the nucleus through nuclear pores (Trus et al. 2004; Cardone et al. 2007). The highly regulated transcription and replication of ILTV DNA occur within the nucleus by utilizing the host cell machinery (Prideaux et al. 1992; Guo et al. 1993). Three classes of genes, namely immediate early (α), early (β), and late (γ) are expressed during the viral transcription and translation process (Honess and Roizman 1974). The non-structural protein products of α genes play a key role in the expression of β genes between 4 to 16 hrs post-infection (Prideaux et al. 1992). The β gene proteins are critical for viral replication and regulate the production of viral structural proteins encoded by late γ genes. The transcription of γ genes takes place 32 hrs post-infection. Nearly 70 virus-coded proteins regulate the viral DNA replication, which includes several enzymes and DNA binding proteins. In the nucleus, the ILTV DNA replication occurs by a rolling circle mechanism with the formation of concatemer, which is cleaved into monomeric units and packaged into preformed nucleocapsids. The formation of viral capsid and packaging of DNA is completed at the end of the viral replication process in the nucleus of the host cell. The nucleocapsids containing DNA acquire an envelope while budding out from the inner lamellae of the nuclear membrane. Subsequently, the virions are transported into the lumen of the endoplasmic reticulum to acquire second envelope and further accumulate within the cytoplasmic vacuoles (Guo et al. 1993). The virions in the cytoplasm associates with the tegument proteins, and get re-enveloped in the trans-Golgi region during second phase of budding. These virions mature in the cytoplasm and are released by either exocytosis or cell lysis (Guo et al. 1993; Mettenleiter 2002).

*In vitro* studies demonstrated that development of progeny virus particle occurs 8 to 12 hrs post-infection and reaches the highest concentration within 24 to 30 hrs post-infection (Davison et al. 1989). After successful replication, the establishment of latency takes place 7-10 days post-infection (Bagust and Johnson 1995). The IR flanking sequences get expressed during latent infections known as latency-associated-transcripts (LATs) are up regulated and maintained until the virus gets reactivated to cause the next episode of cytolytic infection (Bagust 1986). Other uncommon features often observed during ILTV replication are formation of tubular structures and large vacuoles containing virions in the infected cytoplasm (Fuchs et al. 2007).

### 2.3. Antigenicity

Although ILTV strains seem to be antigenically similar based on various assays like immunofluorescence test, virus-neutralization and cross-protection studies (Cover and Benton 1958; Shibley et al. 1962), the difference in virulence has been demonstrated in chicken embryos and in cell culture (Pulsford and Stokes 1953; Jordan 1958; Izuchi and Hasegawa 1982; Russell and Turner 1983). The envelop glycoproteins of ILTV seem to be the potent immunogenic protein capable of stimulating humoral as well as cell mediated immune responses in chicken (York and Fahey 1990). The antigens of ILTV include glycoproteins such as gB, gC, gD, gE, gG, gH, gI, gJ, gK, gL and gM, and are reported to play a crucial role in virus entry and replication (Goraya et al. 2017). Among envelop glycoproteins, glycoprotein G (gG) is identified to facilitate virus entry (Tran et al. 2000), cell-to-cell spread (Nakamichi et al. 2002), and functions as a broad-spectrum viral chemokine binding protein (vCKBP). The gG binds to chemokines of the subfamily C, CC and CXC, and hence prevent the interaction between chemokines and their receptors. It also blocks binding of chemokine to glycosaminoglycans, which is necessary for *in vivo* chemokine activity (Bryant et al. 2003). The vCKBP of ILTV (gG), during early stages of infection, induces innate immune responses by recruiting particular subsets of immune cells (Devlin et al. 2010).

### 2.4. Physico-chemical properties

Given the enveloped nature of the virus, the infectivity of ILTV is greatly modulated by organic solvents such as chloroform, ether and oxidizing agents like H_2_O_2_ (Fitzgerald and Hanson 1963; Neighbor et al. 1994). The sensitivity of ILTV to the temperature differs greatly between its strains. In respiratory exudates and chicken carcasses, the virus can remain infective for 10 days to 3 months at a temperature range of 13-23 °C. The survivability of the virus can be extended for several months when stored at 4 °C in enrichment media like nutrient and glycerol broth. Previous studies revealed the loss of infectivity of ILTV by heating at 55 °C for 15 minutes or 38 °C for 48 hrs while some strains are resistant to heat (Meulemans and Halen 1978). In deep litter, the ILTV survives for 3-20 days at 11-24.5 °C, in the droppings of battery cages for 3 days at 11–19.5 °C and at least for 3 weeks in buried carcasses. The studies demonstrate that the viability of the virus in litter reduces while applying heat at 38 °C for 24 hrs or composting (Giambrone et al. 2008). The virus gets readily destroyed (<1 min) by common disinfectants like 3% cresol, 5% phenol or a 1% sodium hydroxide solution (Meulemans and Halen 1978), however the presence of organic matter affects the efficiency of disinfectants (Ruano et al. 2001).

### 2.5. Host

ILTV has got a narrow host range in contrast to other members of alphaherpesviruses. The main natural host of ILTV is chicken, however, the infections are also reported in peacocks, pheasants, turkeys and guinea fowl (Crawshaw and Boycott 1982; Bautista 2003). Though ducks are refractory to ILT infection, they can act as carriers (Yamada et al. 1980). Other domestic and feral birds such as quail, guinea fowl, pigeons, starlings, sparrows, crows, and doves appear to be resistant to the disease (Beach 1931; Brandly and Bushnell 1934).

### 2.6. Transmission

**Figure 3.**
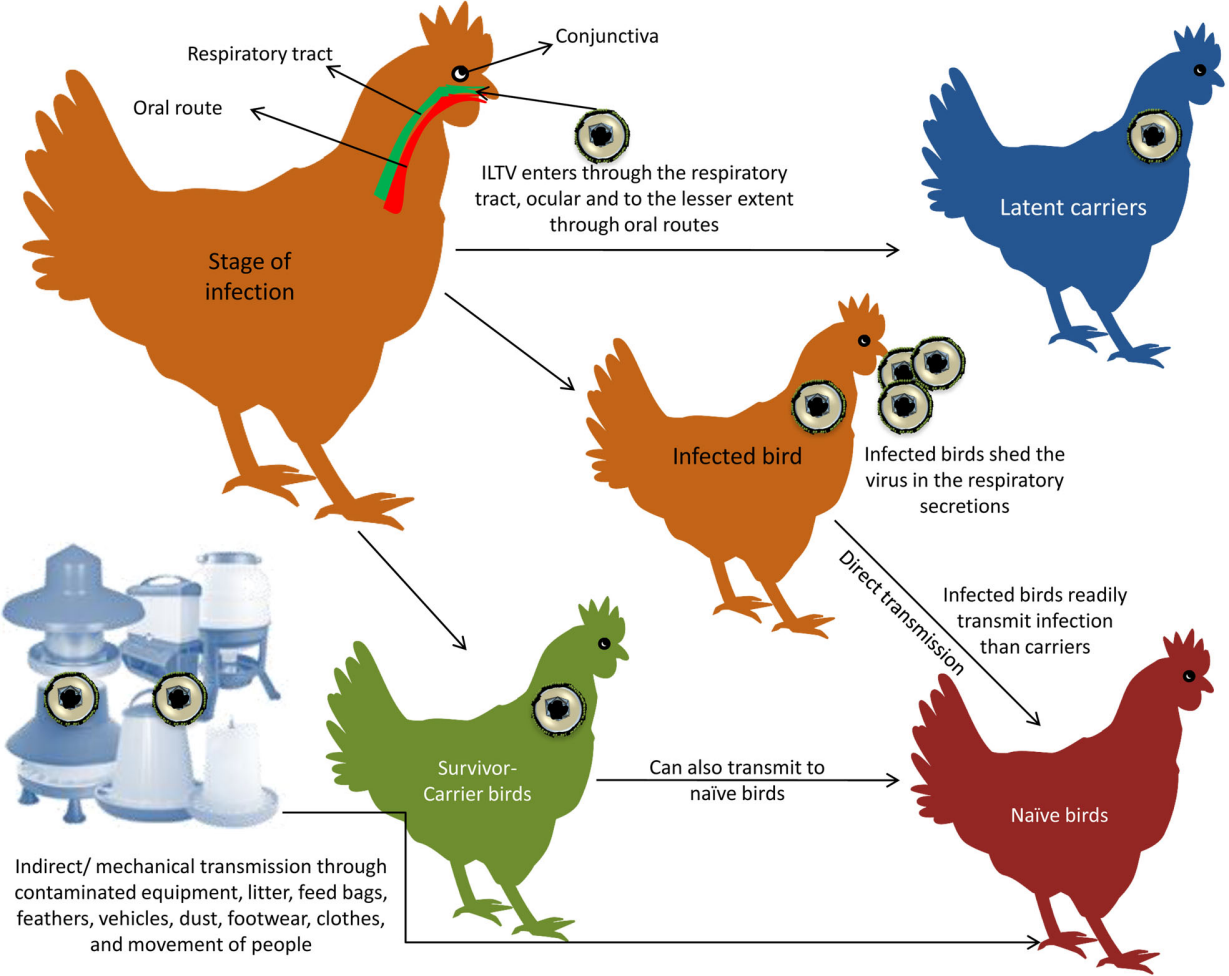
Transmission pattern of ILT virus.

Infected birds shed the virus in their respiratory secretions for 10 days post-infection. ILTV enters into the host through the respiratory tract, ocular and to a lesser extent through oral routes (Figure 3) (Hitchner et al. 1977; Robertson and Egerton 1981; Bagust 1986; Williams et al. 1992). Direct bird-to-bird transmission is rampant in comparison to contact with latently infected or carrier birds. Mixing of vaccinated and naive chickens is important with respect to direct transmission. Neither vertical transmission nor transmission of virus through the egg shell has been demonstrated. No typical viremia during ILTV infection occurs, although spread of the virus to non-respiratory sites has been attributed to infected leucocytes (Chang et al. 1973; Oldoni et al. 2009). Carrier birds that have survived from previous outbreaks also act as a source of infection to the naive birds. The infected birds readily transmit the disease through the oral secretion as compared to clinically recovered birds or latent carriers (Hughes et al. 1987). The virus usually gets introduced into a flock by direct contact with respiratory exudates or indirect/mechanical transmission of contaminated equipment, litter, feed bags, feathers, vehicles, dust, footwear, clothes, and movement of people (Dobson 1935; Beaudette 1937; Mallinson et al. 1981; Zellen et al. 1984). Recent studies demonstrated that ILTV can persist in the biofilm of drinking water lines and spread to susceptible birds (Ou et al. 2011). Darkling beetles and mealworms also act as a source of infection to the birds and the live virus has been demonstrated in darkling beetles even 42 days after the disease outbreak (Ou and Giambrone 2012). Dogs and cats retrieving dead bird carcasses from affected poultry houses also spread the infection (Kingsbury and Jungherr 1958). Wind-borne transmission of ILTV has been demonstrated between commercial poultry operations (Johnson et al. 2005).

## 3. Epidemiology

The disease was first reported in 1925 in the USA (May and Thittsler 1925) and subsequently in Australia, the UK, and Europe (Cover 1996). Veterinarians initially referred to the disease as avian diphtheria, however, the name ILT was adopted in the year 1931 by the special committee of poultry diseases of American Veterinary Medical Association (Guy and Garcia 2008). ILT was the first poultry viral disease for which vaccine was employed based on the cloacal administration (Gibbs 1934). Presently, ILT has been reported in most of the countries worldwide and remains an important disease. The outbreaks are reported in the USA (Dormitorio et al. 2013), Canada (Ojkic et al. 2006), Brazil (Parra et al. 2015), Europe (Neff et al. 2008), Australia (Agnew-Crumpton et al. 2016), China (Zhuang et al. 2014), Egypt (Magouz et al. 2018) and South Asia (Gowthaman et al. 2016). During the period of 2000-2013, the disease had been reported at least in 100 countries (Menendez et al. 2014). Recently, ILTV was confirmed by molecular techniques in Al-Diwaniyah province, Iraq which was the first report from the country (Alaraji et al. 2019). In 2018, three outbreaks of ILT were reported in Windhoek, Namibia causing huge mortality in commercial layers and broilers (Molini et al. 2019). The trend toward high flock density, shorter production cycles, raising of multi-age and multipurpose chicken within same geographical area, and improper vaccination and breach in the biosecurity have contributed to the increased ILT outbreaks across the world (Garcia et al. 2013; Blakey et al. 2019).

ILT remains a serious threat and negatively impacts the poultry industry worldwide since its report in the mid-1920s. Birds of all ages starting from eight days to four years of age (Kingsbury and Jungherr 1958; Jordan 1966; Linares et al. 1994) are susceptible to ILTV infection; however, birds over three weeks of age are reported to be highly susceptible (Dufour-Zavala 2008). High intense poultry rearing, mixing of the different type of birds in the same geographical area and a breach in biosecurity often lead to outbreaks of ILT in many parts of the world. The morbidity and mortality vary depending on the virulence of circulating field strains of ILTV (Devlin et al. 2006; Oldoni et al. 2009), viral load and concurrent infections with other respiratory pathogens (Guy and Garcia 2008). Concomitant respiratory diseases such as *Mycoplasma gallisepticum*, *Mycoplasma synoviae*, infectious coryza, other immunosuppressive diseases such as mycotoxicosis, Chicken anaemia virus, Reticuloendotheliosis virus and Marek’s disease virus-induced immunosuppression, possibly exacerbates the impact of ILT in the field (Zavala 2011). Sporadic cases of ILTV may occur in inadequately vaccinated flocks either due to errors in the application of its vaccines or due to biosecurity failures. In multi-aged layer farms, inadequately vaccinated flocks may get exposed to ILTV during the introduction of younger vaccinated flocks into the farm (Hidalgo 2003). The severe epizootic form is characterized by a rapid spread with a high morbidity (90–100%) or variable mortality ranging from 5 to 70% (average of 10-20%) (Hinshaw et al. 1931; Seddon and Hart 1935). The mild epizootic form is characterized by low morbidity (<5%) to very low mortality (0.1-2%) (Raggi et al. 1961). The vaccine and field strains of ILTV evolve as virulent in high dense poultry rearing areas due to the existence of continuous reservoir, subsequently, the same reverent viruses get established in the field and cause outbreaks (Guy et al. 1990; Kotiw et al. 1995). High-density poultry-producing regions often experience huge economic loss with an overall mortality reaching up to 70% (Bagust et al. 2000). It has been reported that areas previously housed infected flocks probably experience more outbreaks than the farms with no history of ILT (Zellen et al. 1984).

The disease has been reported from several Asian (China, Georgia, India, Japan, Lebanon, Myanmar, Philippines, Sabah, Sarawak, Taiwan, Iraq and Uzbekistan), African (Cameroon, Uganda, Namibia, Egypt, Nigeria), North American (Canada, Delaware, Georgia, Mexico, Maryland, New Brunswick, North Carolina, Ontario, Pennsylvania and Virginia), Central American and Caribbean (Costa Rica, Trinidad and Tobago), South American (Argentina, Brazil, Chile, Peru, Suriname and Uruguay), European (Austria, Belgium, Denmark, Germany, Italy, Moldova, Norway, the Netherlands, Poland, Sweden, Switzerland and UK) and Oceania countries (Australia, Cook Islands, French Polynesia, Guam, Kiribati and New Zealand (Hidalgo 2003; Chacón and Ferreira 2009; OIE 2014; Magouz et al. 2018; Alaraji et al. 2019; Molini et al. 2019). Very recently, it was reported that recombinant ILT virus and CEO vaccine-like virus are causing outbreaks in Eygpt (Bayoumi et al. 2020).

Like other herpes viruses, ILTV can establish latency in the trigeminal ganglion of the central nervous system after 7 days of acute infection (Hughes et al. 1991; Williams et al. 1992). The virus gets reactivated under the stress conditions during shifting, onset of laying and mixing of flocks (Hughes et al. 1989). In general, inapparent, sporadic reactivations with productive replication in the tracheal epithelium lead to virus shedding and transmission of infection to susceptible birds (Bagust and Johnson 1995). Earlier studies demonstrated that detection of long-term tracheal carriers (approximately 2%) among convalescent birds recovered from acute ILT infection play a major role in the establishment of latency (Hanson and Hanson 1984). Recent experimental studies revealed sustained detection of ILTV genome in the Harderian gland, trachea, lung and kidney up to 28 days post-infection (Roy et al. 2015). Backyard poultry flocks also act as an important source of infection for commercial poultry flocks because of viral latency (Ojkic et al. 2006; Kirkpatrick et al. 2006; Neff et al. 2008). Research also reveals a high seroprevalence of ILTV (72%) in non-vaccinated flocks suggesting the role of backyard poultry in its epidemiology (Hernandez-Divers et al. 2008).

Latent infected birds are usually identified by tracheal organ culture and detection of ILTV DNA in the trigeminal ganglion by PCR (Bagust 1986).

## 4. Pathogenesis

The natural portal of entry of ILTV is respiratory and ocular routes. The initial replication takes place in the epithelium of the conjunctiva, respiratory sinuses, larynx and upper respiratory tract to a greater extent (Guy and Bagust 2003). At the primary virus replication sites, the virus titre peaks between 4 and 6 days post-infection, and the virus can be detected in the latency sites Trigeminal ganglion (TRG) from two of cytolytic infections onwards (Bagust 1986; Kirkpatrick et al. 2006; Oldoni et al. 2009). The active cytolytic infection of ILTV results in severe damage to tracheal and conjunctival epithelial lining leading to haemorrhages and other clinicopathological manifestations in birds (Bang and Bang 1967; Tully 1995; Guy and Bagust 2003). Subsequently, the ILTV disseminates to the underlying lamina propria of the tracheal epithelium after invading through the basement membrane with the help of up-regulated cellular proteases (Glorieux et al. 2009; Steukers et al. 2012, Reddy et al. 2014) and reaches to the liver, caecal tonsils and cloaca (Bagust 1986; Oldoni et al. 2009). However, the mechanism of dissemination is not clear. The highest viral titers have been detected in tracheal tissues during 4 to 6 days post-infection and remain in tracheal secretions between 6 to 10 days post-infection (Purcell and McFerran 1969; Hitchner et al. 1977; Robertson and Egerton 1981; Bagust 1986).

The virus replication leads to up-regulation of genes related to cell growth and proliferation. The infected cells produce cytokines and other inflammatory mediators leading to immune responses such as elevated body temperature, intensive edema, and infiltration of lymphocytes (Purcell 1971a; Guy and Garcia 2008). Scattering of CD4+ and CD8+ cells, as well as clustering of B lymphocytes in the mucosa, were detected in ILTV infection (Devlin et al. 2010). At this stage, the outcome of infection is influenced by the type of inflammatory cells and the ability to establish adaptive immune response. ILTV establishes latency in the trigeminal ganglion corresponding to the induction of effective adaptive immunity following the lytic phase of an infection (Williams et al. 1992). Reactivation of ILTV from latency is mediated by thymidine kinase and infected-cell polypeptide 4 (ICP4) (Johnson et al. 1995; Schnitzlein et al. 1995; Han et al. 2002).

## 5. The disease

### 5.1. Clinical signs

The incubation period of ILTV varies between 6 and 14 days (Kernohan 1931; Seddon and Hart 1935). Previous experimental studies showed that ILTV shedding started 2 days post-infection and 4 days before the appearance of clinical signs (Davison et al. 1989). The clinical course of ILT varies from 11 days to 6 weeks depending on the form of the disease (McMullin 2004). The clinical signs are characterized by a sudden increase in average daily mortality in the affected flock (Aziz 2010). The severity of the disease is influenced by the virulence of the virus, stress conditions, co-infections with other pathogens, immune status of the flock and age of the birds (Gowthaman et al. 2016). The infection is characterized by peracute, acute and chronic forms of ILT.

#### 5.1.1. Peracute form

**Figure 4.**
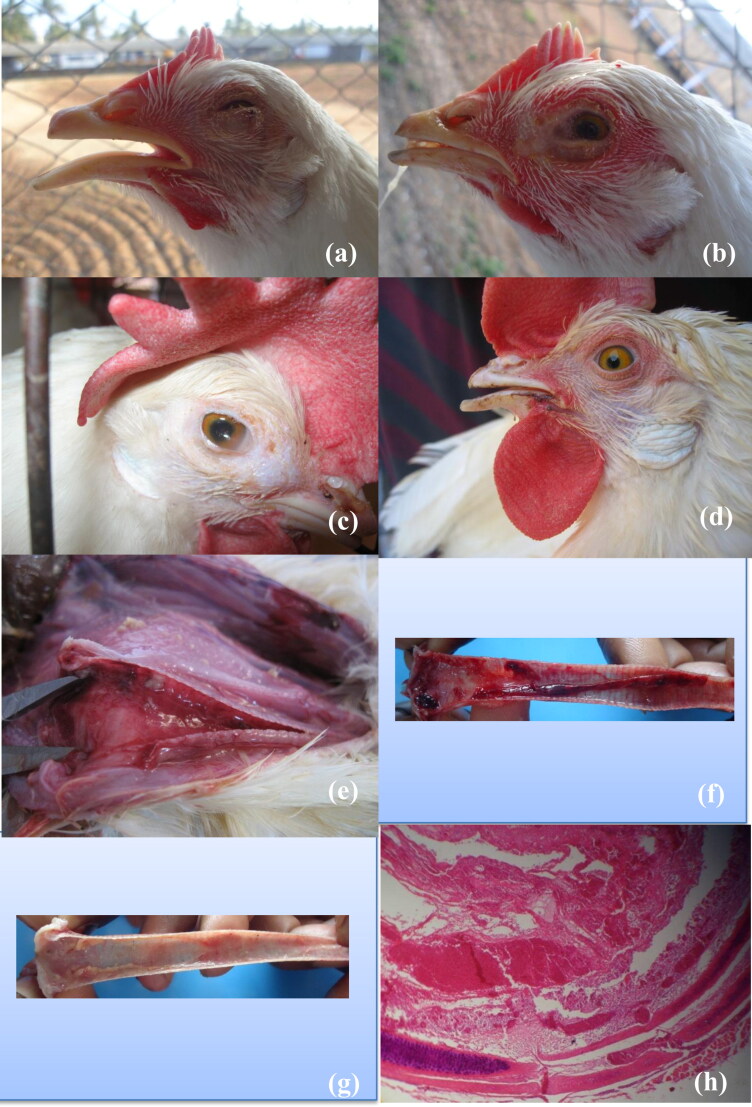
Different clinico-pathological manifestations of ILTV infection: a. Acutely infected bird shows severe gasping. b. Oculo-nasal discharges in early stages of infection. c. Facial swelling and persistent oophoria in sub-acute to chronic stage of ILTV infection. d. Dried bloody exudates on the commissure of the mouth. e. Fibrino-haemorrhagic exudates in the lumen of the trachea. f. Blood clots in the lumen of the trachea in acute form of ILT. g. Pseudomembrane formation in chronic form of ILT. h. Diffuse hemorrhagic inflammation of trachea lading to accumulation/obstruction of tracheal lumen with fibrino-haemorrhagic and necrotic tissue debris.

It is characterized by sudden onset of rapid spread and high mortality which may exceed 50% (OIE 2014). The affected birds become lethargic, often exhibit moderate-to-severe conjunctivitis with swollen eyelids and increased lacrimation. Sometimes death may occur in birds with good body condition before the appearance of any clinical signs (Preis et al. 2013). The clinical signs (Figure 4) are characterized by dyspnea and gasping with an extension of the head and neck. Coughing, rattling, and gurgling also noticed when the birds try to expel the clotted blood and debris from the obstructed trachea (Guy et al. 1990; Blakey et al. 2019). The clotted blood is also found in cages, feed turfs, walls and floor of the poultry houses. The affected birds usually die within 3 days (Cover 1996).

#### 5.1.2. Acute form

Characteristic dyspnea is commonly noticed in the acute form of ILT, but the onset is not sudden or severe as seen in peracute form. Initially, the affected birds become inactive and exhibit anorexia (Guy and Bagust 2003). The internal core temperature increases between 4 and 6 days post-infection, and the total leukocyte count shows mild to marked lymphopenia and heterophilia (Chang et al. 1997). Tracheal obstruction with clotted blood and exudates results in a long drawn out gasps with open-mouthed breathing, high-pitched squawk and moist rales (Kernohan 1931; Jordan 1958). The affected birds may also show purulent conjunctivitis with frothy exudates in the inner canthus of the eye, sinusitis and nasal discharge (Beach 1926). The morbidity may reach 100% and the mortality varies from 10 to 30%, which may last up to 15 days. Varying level of egg production is noticed in layer flocks, some flocks may experience the complete cessation of egg production, which may recover to the normal level in due course of time (Lohr 1977; Creelan et al. 2006).

#### 5.1.3. Chronic form

The mild or chronic ILT resembles with other respiratory infections characterized by unthriftiness, coughing, moist rales, head shaking, squinting eyes, swelling of the infraorbital sinuses (almond-shaped eyes), drop in egg production (up to 10%), and reduced body weight (Hinshaw et al. 1931; Ou et al. 2012). The morbidity may go up to 5% and mortality usually restricted <2% (Bagust et al. 2000).

### 5.2. Gross lesions

The gross lesions are usually restricted to sinuses and upper respiratory tract and vary with the severity of the disease (Seifried 1931; Gough et al. 1977). The gross lesions in peracute form consist of mucoid rhinitis and haemorrhagic tracheitis with blood clots (Guy and Bagust 2003; Barhoom and Dalab 2012). Yellow caseous exudates (cheesy plug) also observed in primary bronchi when the lesions extend deeply (OIE 2014). In the acute form, yellow caseous diphtheritic membranes adherent to the larynx and mucosa of the upper trachea with or without haemorrhages are commonly noticed (Gowthaman et al. 2014). The membrane also forms obstructive plugs in the larynx and syrinx regions leading to suffocation and death. Excess mucous with or without diphtheritic exudates may be observed in the tracheal lumen in the chronic or mild form of ILT (Linares et al. 1994). A pseudomembrane formation with fibrino-necrotic exudates adhering to the upper respiratory tract can also be noticed (Russell and Turner 1983; Russell 1983). Apart from tracheal involvement, conjunctivitis is characterized by edema and congestion with increased ocular discharge (Hinshaw et al. 1931; Kirkpatrick et al. 2006). The inflammatory response in nares is characterized by heterophilic exudates (Gowthaman et al. 2014). The involvement of lungs and air sacs are rare. However, congestion of the lungs and thickening of air sacs with caseous exudates in the lumen are occasionally seen (Aziz 2010). In concurrently infected cases, lesions such as muco-fibrino acute rhinitis and sinusitis, occlusion of paranasal sinuses by caseous exudate, facial swelling, and muco-fibrino tracheitis have been observed (Couto et al. 2015). Recently, a solitary case of severe erosive esophagitis and pharyngitis accompanied with epithelial degeneration, necrosis, and syncytia formation with intranuclear inclusion bodies has been reported as an atypical ILT (Sary et al. 2017).

### 5.3. Microscopic lesions

**Figure 5.**
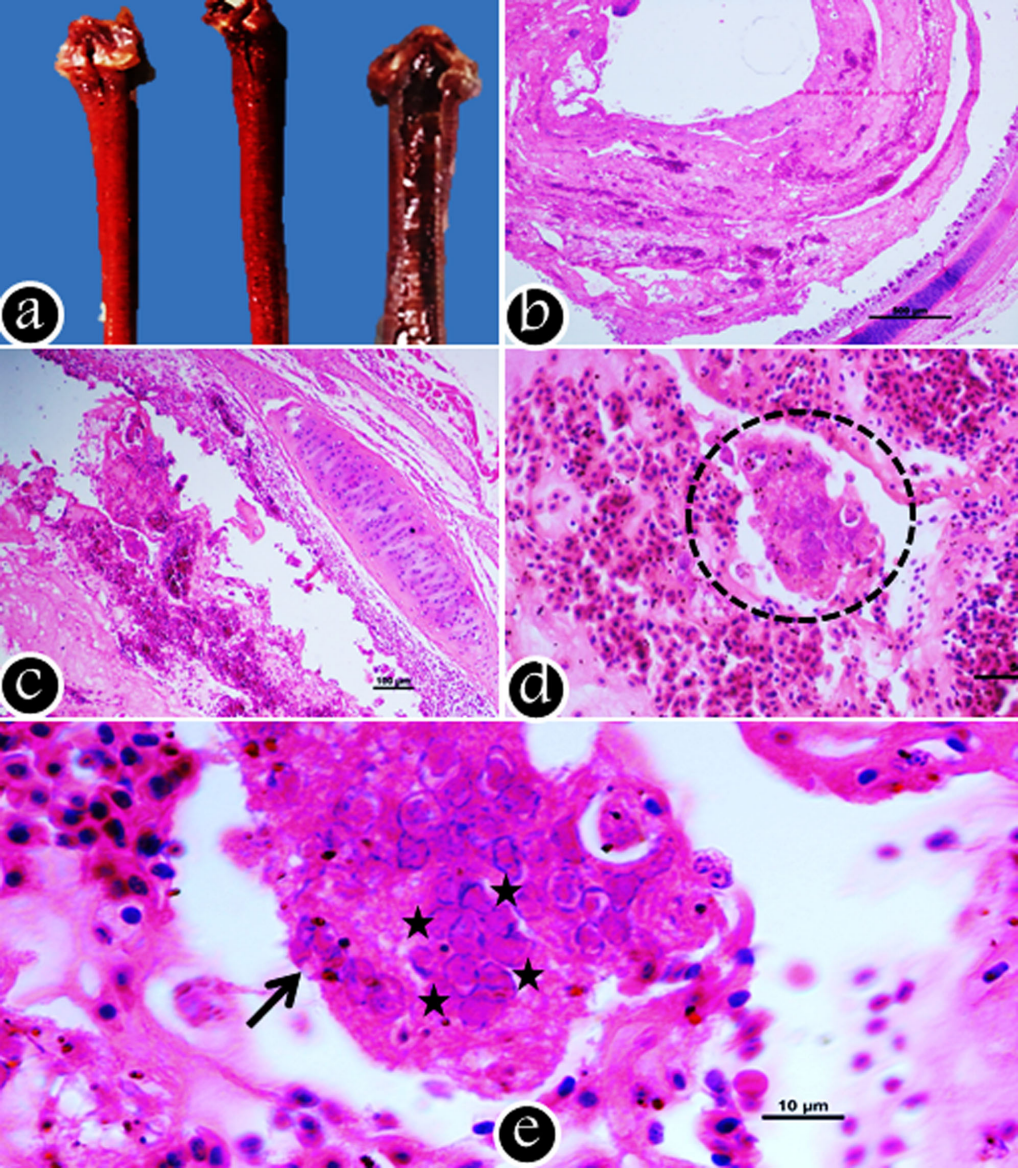
Gross and Histopathology: (a) Severely congested and hemorrhagic trachea collected from field ILT outbreaks; (b) Cross section of trachea showing intraluminal accumulation of necrotic debris mixed with fibrino-heterophilic exudates (H&E, 4X); (c) Section of trachea showing denudation of mucosal layer, mucosal hemorrhages amidst marked fibrinous exudation (H&E, 10X); (d) Sloughed of tracheal mucosa showing a severe hemorrhages and large multinucleated syncytia (circle) (H&E, 20X); Higher magnification of syncytia (arrow) showing presence of intranuclear eosinophilic inclusion bodies (star marks) (H&E, 100X).

The microscopic lesions are restricted to the conjunctiva, sinuses, trachea, and lungs (Linares et al. 1994). In conjunctiva, they consist of early hyperemia, swelling, infiltration of inflammatory cells, followed by epithelial damage. This further leads to sloughing of conjunctival epithelium with an accumulation of inflammatory exudates primarily containing red and white blood cells and fibrinocellular debris (Figure 4) (Aziz 2010). The initial microscopic changes in trachea include infiltration of inflammatory cells. The infected epithelial cells undergo hyperplastic changes followed by lymphocytic and histiocytic infiltrations in the mucosa and sub-mucosa as the disease advances (Russell 1983). Subsequently, the tracheal epithelial cells undergo necrosis with diffuse denudation that results in protrusion and rupture of blood vessels of lamina propria into tracheal lumen leading to severe laryngitis and tracheitis (Sary et al. 2017). Intranuclear basophilic or eosinophilic inclusion bodies surrounded by a halo are usually seen during initial stages of infection (1–5 days) and disappear later due to necrosis and denudation of epithelial cells (Seifried 1931; Guy et al. 1992; Vanderkop 1993). During this stage, the lumen of the trachea contains varying amount of exudates with fibrin, inflammatory cells, red blood cells, epithelial debris and syncytial cells with or without intranuclear inclusion bodies (Hayashi et al. 1985). Regeneration starts six-days after infection with the proliferation of the remaining basal cells in birds that survive the acute phase (Bagust et al. 2000). Subacute hyperplastic tracheitis characterized by proliferation of several layers of regenerating, undifferentiated, non-ciliated epithelial cells lining the mucosa and mucous glands become evident during the healing stage. The histopathological changes in primary and secondary bronchi are characterized by epithelial degeneration and denudation with infiltration of mononuclear cells (Preis et al. 2013).The syncytial cells with the intranuclear inclusion bodies may also be seen in the lesions (Purcell 1971b; Timurkaan et al. 2003). Gross and histopathology of lesions of ILTV are depicted in Figure 5.

## 6. Diagnosis

Infectious laryngotracheitis in chicken can be tentatively diagnosed based on the clinical signs such as conjunctivitis, gasping, open mouth or extended head respiration, expectoration of bloody mucous, dyspnoea, and finding lesions including catarrhal to hemorrhagic tracheitis, fibrinopurulent to caseous exudates or cheesy or caseous plugs in the larynx and trachea on necropsy. The suspected cases are subjected to laboratory diagnosis by conventional and molecular diagnostic tests. The conventional methods include histopathology, virus isolation by embryonated chicken eggs and cell culture, immunofluorescence (IF), immunoperoxidase (IP) assay, and serology (Burnet 1934; Wilks and Kogan 1979; Hughes and Jones 1988; Guy et al. 1992; Godoy et al. 2013). Detection of syncytial cells and intranuclear inclusion bodies in the trachea, eyelid, and lung tissues using histopathology is routinely practiced (Humberd et al. 2002; Timurkaan et al. 2003; Srinivasan et al. 2012).

The preferred clinical samples for isolation of ILTV are conjunctiva, larynx, trachea, lung and their exudates. Among the different clinical materials, lungs, tracheal scrapings, and exudates from trachea are ideal for virus isolation (Tripathy and Garcia 1998). The ILTV is usually isolated and propagated in 9-11 days-old embryonated chicken eggs through chorioallantoic membrane (CAM) inoculation. Opaque plaques can be observed in ILTV infected CAM as early as 48 h post-inoculation and embryo death occurs between 2 and 8 days post-infection. The embryonic survival time increases with subsequent additional egg passages leading to effective replication of the virus to significant titers (Garcia and Riblet 2001).

The number of primary avian cell cultures, including chicken embryo liver (CEL), chicken embryo lung, chicken embryo kidney (CEK), and chicken kidney (CK) cell cultures are commonly used for ILTV isolation (Chang et al. 1977; Meulemans and Halen 1978; McNulty et al. 1985; Hughes and Jones 1988; Schnitzlein et al. 1995). The cell culture method is more economical and rapid than egg inoculation. The sensitivity of the isolation and virus yield are influenced by the type of cell cultures. The CEL is found to be the most sensitive for ILTV isolation followed by CK. The CEK, chicken embryo lung, chicken embryo fibroblast, Vero, and quail cells were found less sensitive to ILTV infection (Hughes and Jones 1988; Garcia et al. 2014). In addition, Leghorn male hepatoma cells were also used to propagate the virus in research laboratories (Schnitzlein et al. 1995). The cytopathic effects of ILTV infection are characterized by the swelling of cells, chromatin displacement, rounding of the nucleoli and syncytia formation. Intranuclear inclusions are detected as early as 12 hrs post-infection, however, the formation of multinucleated giant cells may be observed 24 hrs post-infection in avian leukocyte cultures (Hinshaw et al. 1931; Chang et al. 1977). The plaque size and morphology are influenced by the strains of ILTV (Srinivasan and Malick 1977; Hughes and Jones 1988). CEK cells infected with ILTV reveal presence of large cytoplasmic vesicles, which become basophilic mass as the cells degenerate (Reynolds et al. 1968). Macrophage culture is equally susceptible to ILTV. However, the viral replication is limited (Calnek et al. 1986). Other cell lines from heterologous hosts such as QT35 or IQ1A from quail-origin, and Vero cells permit limited replication but with a very low virus titre even after several passages. Other culture systems routinely used are tracheal organ culture (TOC) and conjunctival organ cultures (COC) obtained from chicken embryos or day old chicks. However, these TOC and COC are utilized to study the host pathogen interaction (Bagust 1986; Jones and Hennion 2008; Reemers et al. 2009).

Apart from virus isolation, the IF, IP, and immunohistochemistry (IHC) can be used to detect the ILTV in tracheal tissues and smears (Ide 1978). The sensitivity of IHC is reported more superior than IF (Hitchner et al. 1977). The distribution of ILTV antigen within different tissues of respiratory tract is highly variable and highest IHC positivity has always been found in trachea than any other organs (Yavuz et al. 2018).

Agar gel immune diffusion (AGID) technique using ILTV hyperimmune serum is commonly used to differentiate it from a diphtheritic form of fowlpox (Fukui et al. 2016). However, the sensitivity was lower when compared with other serological techniques like virus neutralization test (Devlin et al. 2011), indirect immunofluorescence test and ELISA (Jordan and Chubb 1962; Godoy et al. 2013). Antigen-capture ELISA (AC-ELISA) using ILTV monoclonal antibodies is applied for rapid and more accurate detection of ILTV than AGID, IF or virus neutralization (York and Fahey 1990). As a gold standard, ELISA is preferred for the detection of antibodies from the field sample. Recently, glycoprotein D (gD) based ELISA has been developed where two immunogenic regions were identified and synthesized. This synthetic peptide was used in the developed ELISA which showed sensitivity of 96.9% and specificity of 87.5% (Kumar et al. 2019).

Although the conventional methods are cost-effective and widely applied in diagnostic laboratories, these methods have some limitations like low sensitivity, labor-intensive and time-consuming. Several molecular-based techniques such as PCR, real-time PCR, nested PCR, restriction fragment length polymorphism (RFLP), *in situ* hybridization have been applied to detect the ILTV because of its high sensitivity, accuracy, rapidity, reproducibility, and simplicity (Nielsen et al. 1998; Vögtlin et al. 1999; Humberd et al. 2002; Creelan et al. 2006; Callison et al. 2007; Mahmoudian et al. 2011; Zhao et al. 2013). Both the probe based and dye based real-time PCR assays and nested real time PCR are found to be highly sensitive as these assays can detect as low as 1^9^ to 1° copies of virus in biological samples (Zhao et al. 2013; Davidson et al. 2015; Santander Parra et al. 2018). Although these molecular methods have significant diagnostic value, they do not discriminate between viable and non-viable virions (Menendez et al. 2014). Hence, positive results need to be carefully interpreted and carryover contamination should be ruled out.

Among different molecular techniques, PCR and quantitative real-time PCR (qRT-PCR) are the widely used and preferred molecular assays for confirmation and quantification of viral load in biological samples due to their higher diagnostic sensitivity and accuracy (Guy et al. 1992; Williams et al. 1992; Scholz et al. 1994; Abbas and Andreasen 1996; Creelan et al. 2006; Kirkpatrick et al. 2006; Fuchs et al. 2007; Chacón and Ferreira 2009; Zhao et al. 2013; OIE 2014; Roy et al. 2015; Santander Parra et al. 2018). Earlier, the wild and vaccine strains of ILTV were differentiated based on restriction length polymorphism (RFLP) profiles (Leib et al. 1986; Andreasen et al. 1990; Keeler et al. 1993; Kotiw et al. 1995; Oldoni and García 2007; Craig et al. 2017). Recently, Fakhri et al. (2019) developed high-resolution melting (HRM) analysis to classify ILTV strains and detect ILTV recombination events during field outbreaks. The recent advances in molecular sequencing technologies enabled rapid identification of genetic variations with high precision. Next generation sequencing (NGS) platforms such as hybrid next generation sequencing (h-NGS) are found to be useful to identify mutations in genes related to high and low virulence. Genomic sequences of low and high passaged CEO and TCO ILTV strains were determined by h-NGS wherein both the CEO and TCO strains expressed variable mutations upon passages in the target host. The common genes mutated in these two strains were ORFC, UL27, UL28, UL39_,_ and the virulent ILTV strains isolated in USA showed frequent Thr644 mutation within UL27 gene. Although the genes responsible for reversion to virulence are not very clear, the gene segment US10 has been identified as one of the potential virulence factors for TCO revertant. Similarly, the gene UL41 had been found to be responsible for robust gain in virulence of CEO strains (Garcia et al. 2013). MinIon sequencing was also used as diagnostic tool in USA to genotype the different ILTV isolates. Full genome (n = 27) of ILTV were analyzed and it was identified to have 9 genotypes which can be grouped into 5 genotypes based on single allele assay using MinIon (Spatz et al. 2019).

Recently, a TaqMan single nucleotide polymorphism genotyping (TaqMan-SNP) assay has been developed to study the ILTV recombination in the natural host. Based on this assay, 11 SNPs within genes UL (-1), US5, US6, US7, US8, US9 and two SNPs in UL43 and UL47 genes were identified, and 67% of the progeny ILT viruses were found to be recombinant (Loncoman et al. 2017).

## 7. Differential diagnosis

The other respiratory diseases exhibiting similar clinical disease must be differentiated from ILT. The diphtheritic lesions induced by ILT spread over the whole length of trachea and resemble lesions induced by the fowlpox virus (Tripathy and Reed 2013). Tracheal lesions in mild or low virulent form of ILTV is similar to that of lesions caused by other respiratory pathogens such as avian influenza virus, Newcastle disease virus, infectious bronchitis virus and fowl adenovirus (Davidson et al. 2015).

### 7.1. Differentiation of field isolates and vaccine strains

Differentiation of field and vaccine strains of ILTV is complicated because of its high antigenic and genetic resemblance (Guy and Garcia 2008). Several methods including chicken embryo virulence test (Izuchi and Hasegawa 1982), restriction endonuclease analysis (Keller et al. 1992), and DNA hybridization assays (Kotiw et al. 1995) have been attempted to differentiate the wild-type and vaccine strains of ILTV. Later, these methods have been replaced by PCR-RFLP of multiple genes and genome regions including ICP4, TK, gE, gG, ORFB-TK, and ICP18.5 to UL43 genes (Neff et al. 2008; Moreno et al. 2010). A recent approach using sequence analysis of ICP4 gene was successfully used to differentiate vaccine and wild-type strains of ILTV (Chacón and Ferreira 2009).

## 8. Vaccination

Good biosecurity practices combined with vaccination are the practical methods to control ILTV in the absence of any effective treatment. Nevertheless, ILTV was the first major poultry disease for which an effective vaccine was introduced (Gibbs 1934). However, the disease remains an important issue in the poultry-dense areas (Couto et al. 2015; Chacón et al. 2015; Yan et al. 2016; Gowthaman et al. 2016). The modified live attenuated ILTV vaccines including CEO and TCO have been used for several decades. Chicken embryo origin live attenuated vaccines were the first commercially used vaccine which were introduced on the market during 1950s and start of 1960s (García and Zavala 2019). The protective efficacy of CEO vaccines is better when compared to TCO vaccines (Andreasen et al. 1989). These vaccines are used for prevention as well as during the phase of an outbreak to control virus spread and shorten its duration (Bagust et al. 2000).

Preventive vaccination of ILTV is given at 6 to 8 weeks of age, followed by the booster at 12 to 15 weeks for layers and breeders (Gingerich and Carver 2006). These vaccines elicit the immune response by causing infection in the trachea without producing disease. The highest protective immunity is attained from 15 to 20 weeks post-vaccination, which may last over a year (Neff et al. 2008) and no interference has been reported between ILT and other vaccines if the vaccine interval is more than 2 weeks (Aston et al. 2019). ILTV vaccination is not suggested for broilers because of its economical concern (Giambrone et al. 2008). The route of vaccine administration has always been critical to ensure protection and avoid adverse vaccine reactions. The eye drop method is considered comparatively safer and gives more protection than mass application methods like drinking water and spray administration. A superior ILTV vaccine must contain a titer of >10^2^ plaque-forming units/ml to induce adequate immunity when delivered by routes other than the oral route (Raggi and Lee 1965). Apart from the effectiveness, CEO and TCO vaccines have undesirable properties of reversal to the virulent form following bird to bird passages leading to vaccinal laryngotracheitis in the field (Dufour-Zavala 2008; Chacón et al. 2015). In some occasions, vaccination leads to the creation of latent carrier birds, which act as a source of infection to unvaccinated flocks (Bagust 1986). These latent viruses are reactivated, leading to intermittent shedding of ILTV when the birds are subjected to stress conditions like onset of lay, transport, vaccination, etc. causing further spread of disease to the susceptible birds (Guy et al. 1990; Hughes et al. 1991). Extensive use of live attenuated vaccines resulted in new outbreaks of ILT in many parts of the world. Previous experimental studies suggested exacerbated prolonged ILT infections following poor mass CEO vaccination (García 2016). Another study showed that CEO vaccinated birds had better protection even at 35 weeks of age when compared with TCO or HVT-LT (Palomino-Tapia et al. 2019).

A study of ILTV outbreaks in different geographical regions of USA revealed that 75% of the ILTV isolated from the field were resulting from CEO vaccine strains (Garcia and Riblet 2001). Recent studies revealed that spontaneous natural recombination between attenuated vaccines in the field leads to the emergence of novel virulent variants of ILTV (Lee et al. 2012; Agnew-Crumpton et al. 2016). Very recently, whole genome analysis of an ILTV isolate in Australia revealed that recombination is a continuous process leading to virulent virus. The isolate was suggested to be a recombinant of vaccine strain and another recombinant virus (Sabir et al. 2020). To overcome the limitations and biosafety concerns of conventional vaccines, recombinant vaccines such as FPV vector vaccine expressing glycoprotein B and UL32 genes of ILTV(McGeoch et al. 2006), two HVT vector vaccines, one containing ILTV glycoproteins I and D, and another containing ILTV glycoprotein B (Esaki et al. 2013), LaSota strain of Newcastle diseases virus (NDV) that expresses ILTV glycoproteins (Kanabagatte Basavarajappa et al. 2014; Zhao et al. 2014), modified very virulent Marek’s disease virus (vvMDV) that express ILTV glycoproteins (Gimeno et al. 2015) and recombinant vaccines expressing different ILTV glycoproteins including gB, gC, gD, gG, gI, gJ, TK, UL0, UL32, and UL47 (Vagnozzi et al. 2012; Coppo et al. 2013; Yu et al. 2017) were introduced and evaluated. NDV vector expressing gD of ILTV protected birds against both ILT and ND. Similarly the construct was stable and safe even after 8 chicken egg passages (Yu et al. 2020). The F gene of NDV, gD and gI genes of ILTV double recombinant HVT vector vaccine ((HVT-NDV-ILT) showed 97%, 94% and 97% protection against velogenic NDV (GB Texas), ILTV (LT 96-3) and Marek’s disease virus (GA 5) strains, respectively (Gergen et al. 2019). The advantages of these recombinant vaccines are lack of transmission, the absence of reversion to a virulent form, and lack of latency (Johnson et al. 2010; Coppo et al. 2013; Coppo et al. 2013; Zhao et al. 2014). Utilizing a bacterial artificial chromosome (BAC), genes encoding glycoprotein B (gB) or glycoprotein J (gJ) of ILTV were introduced into meq gene deleted very virulent MDV (vvMDV) to create the BACDMEQ-gB and BACDMEQ- gJ recombinant strains, and the resulted BACDMEQ-gB recombinant had conferred immunity after subcutaneous vaccination at day one after hatch which was comparable to commercial ILT-HVT vectored vaccine (Gimeno et al. 2015; García and Zavala 2019). A study was conducted using the recombinant herpesvirus of turkey based ILT vaccine rHVT-LT and CEO ILT vaccine to know the effect of combined vaccine, to know the effect of rHVT-LT on CEO vaccine and protective efficacy of the vaccine. Results showed that birds primed with rHVT-LT followed by booster with CEO showed reduction in replication of CEO virus and protection was good in combined vaccine compared with rHVT-LT alone (Maekawa et al. 2019). Another study showed that *in ovo* vaccination of rHVT-LT did not stop the challenge virus spread to naive birds (Maekawa et al. 2019). Eye drop vaccination of CEO based ILT vaccine showed that conjunctiva-associated lymphoid tissues (CALT) and Harderian gland (HG) had a strong role in development of immunity against ILTV (Beltrán et al. 2017). Virus like particles (VLPs) carrying glycoproteins B (gB) or G (gG) had been recently developed and were studied by administration through *in ovo* and intra muscular route. VLP-gG *in ovo* vaccination produced antibody response and there was no side effect due to *in ovo* vaccination. Hence *in ovo* vaccination using VLPs can be a promising option for control of ILT (Schädler et al. 2019).

These vaccines are administered *in ovo* at day 18^th^ of embryonating period or subcutaneous route during one day of age. Although the recombinant vaccines have the advantages over conventional vaccines, they fail to give sterile immunity (Johnson et al. 2010; Vagnozzi et al. 2012), moreover protection against ILT was severely affected when ILT and IBD products were inserted into separate HVT vectors (Dunn et al. 2018), because of competition for replication. Hence, it is necessary to develop duel insert vaccines to overcome this disadvantage. Studies have been attempted to develop new ILTV vaccines using deletion of genes such as TK (Han et al. 2002), UL0 (Veits et al. 2003), gJ (Fuchs et al. 2005), gG (Devlin et al. 2006), UL47 (Helferich et al. 2007) and gC (Pavlova et al. 2010). Most recently, Ali et al. (2019) analysed nine epitopes as promising vaccine candidate against ILTV which included 3 B cell epitopes (190KKLP193, 386YSSTHVRS393, and 317KESV320) and six T cell epitopes which comprised three MHC-I binding epitopes (118YVFNVTLYY126, 335VSYKNSYHF343, and 622YLLYEDYTF630) and three MHC-II binding epitopes (301FLTDEQFTI309, 277FLEIANYQV285, and 743IASFLSNPF751). Though these findings are promising, it needs further studies before commercially introducing it into poultry industry. As on today HVT and fowl pox vector based recombinant ILT vaccines are available in the market. The novel approaches that are independent of the immune system of the host, including a high level of biosecurity, exploration of host genetic resistance and further improvement of novel vaccines are needed to control ILTV outbreaks.

## 9. Control and eradication

ILT remains a significant disease in all intensive poultry producing regions of the world. The eradication of ILTV requires an implementation of coordinated control programme with the cooperative effort of government agencies, laboratories, poultry producers, poultry health companies, and veterinarians (Dufour-Zavala 2008). The control measures should be focused on timely diagnosis, implementation of strict biosecurity, cleaning, and disinfection, application of geographic information system (GIS) technology, vaccination, and communication between poultry farmers and control agencies (Mallinson et al. 1981; Guy and Garcia 2008). There are few reports regarding the use of herbal drugs for the treatment of ILTV. At higher concentration, Yinhuangerchen, a Chinese herbal mixture reduced the level of ILTV in tissues and also developed mucosal immunity, in birds treated with Yinhuangerchen mixture after 72 hours post-infection (Zhang et al. 2018). Cheng et al. (2011) found that the *Huangqi Maxingshigan* decoction, containing five herbal medicines (*Almond*, *Gypsum fibrosum*, *Herba ephedrae*, *Radix astragali*, *Radix glycytthizae*) provided an antioxidant defense in the process of anti-ILT. Additionally, it can enhance mucosal immunity through induction of sIgA production.

An effective biosecurity plan includes site quarantine and hygiene, restriction of movement of potentially contaminated workers, equipment, feed, vehicles, and birds. Proper disinfectant and litter decontamination should be taken into consideration. Preventive measures should also focus on the control of feral birds, rodents, dogs and cats accessing the barns (Volkova et al. 2012). The dead birds should be properly removed and disposed of safely. Proper cleaning and disinfection of poultry houses should be carried out and the downtime should be extended between subsequent batches. The backyard and fancy chicken flocks should be closely monitored and included in the eradication plan since they may act as reservoirs of ILTV (Mallinson et al. 1981). Further, the virus spread and length of an outbreak can be reduced by therapeutic vaccination. The maximum level of diversity in the ILTV progenies was associated with increased frequency of recombination in the ‘hot-spot’ regions of the virus genome (Loncoman et al. 2017).

## 10. Conclusions and future prospects

Rapid expansion of poultry population has led to increased outbreaks of ILT in many poultry producing regions of the world particularly in countries with high poultry density. Being a Gallid herpesvirus 1, ILTV possesses all the common features of other herpes viruses such as latency and carrier status. Since its first detection in 1925 in USA, ILTV became well established in poultry populations where CEO origin vaccine has been intensively used as a part of control programme. Like other herpesviruses, ILTV undergoes latency in trigeminal ganglion and get reactivated whenever the birds undergo stress leading to increased shedding and environmental spread which makes eradication of ILTV difficult. The darkling beetles acts as an important carrier of ILTV in poultry environments. Further, secondary infections increase the severity of the clinical disease and economic losses. Extensive research have resulted in increased understanding of herpesvirus transmission, pathogenesis and control. This knowledge helps to reduce the impact of ILTV in poultry industry in near future.

Virus isolation, serological techniques and histopathology have been commonly used to diagnose the disease. Modern diagnostic techniques such as PCR, PCR-RFLP, Real time PCR, and NGS have been commonly applied to understand the epidemiology of ILT outbreaks. Increased use of CEO vaccines without much biosecurity leads to outbreaks and persistence of vaccinal ILT worldwide. Recombinant vaccines have been developed by expressing ILTV surface glycoproteins in vectors such as HVT, NDV and Fowl pox virus as an alternative control strategy. Though vectored vaccines show some protection, they are not fully successful in controlling ILT outbreaks. Hence, more sophisticated vaccines need to be developed for ILTV by using advanced biotechnological tools including reverse genetics, recombinant DNA technology with use of novel adjuvants and exploiting advanced delivery methods by overcoming the disadvantages of commercially available vaccines. Besides vaccination, reducing stress conditions, adapting strict biosecurity measures and implementing appropriate pest control programmes are important in ILTV control programme to be made more effective. Of note, certain herbal extracts have been found promising in reducing the disease severity.

ILT remains a significant threat to the poultry industry worldwide. Improved understanding of the virus biology, epidemiology, and pathogenesis along with strict biosecurity may help to control the disease outbreaks. The coordinated plan including rapid diagnosis, implementing strict biosecurity, the vaccination programme, use of GIS technology, proper cleaning, disinfection and heating of poultry houses and increased communication between government and industry will be the most effective approach in controlling ILTV.
